# Supplementary material for: KD-64—A new selective A2A adenosine receptor antagonist has anti-inflammatory activity but contrary to the non-selective antagonist—Caffeine does not reduce diet-induced obesity in mice
Source: PLoS One. 2020 Jun 18;15(6):e0229806. doi: 10.1371/journal.pone.0229806 (PMC7302451; doi:10.1371/journal.pone.0229806)
Supplement: Attachment — Submitted filename: Response to Reviewers.docx [file pone.0229806.s001.docx]

Journal Requirements:

When submitting your revision, we need you to address these additional requirements.

1. Please ensure that your manuscript meets PLOS ONE's style requirements, including those for file naming. The PLOS ONE style templates can be found at

http://www.journals.plos.org/plosone/s/file?id=wjVg/PLOSOne_formatting_sample_main_body.pdf and http://www.journals.plos.org/plosone/s/file?id=ba62/PLOSOne_formatting_sample_title_authors_affiliations.pdf

Manuscript meets PLOS ONE's style requirements, including those for file naming.

2. At this time, we request that you please report additional details in your Methods section regarding animal care,: a) Please include the source of the animals used in this study and  b) please describe any steps taken to minimize animal suffering and distress, such as by administering anesthetics or analgesics.

Source of the animals used in this study has been included. **LINES: 138-140**

Following sentence has been added: “The animals were obtained from the Animal House of the Faculty of Pharmacy of the Jagiellonian University Medical College.

Steps taken to minimize animal suffering and distress have been described. **LINES: 147-155**

Following paragraph has been added:

“During the experiments, when possible, steps were taken to minimize animal suffering and distress. Unfortunately, in the anti-inflammatory studies, it was not possible to administer any anaesthetics and analgesics since they would interfere with the tests, however the animals were not isolated individually to avoid developing stress. In the studies of anti-obesity properties efforts were made to create the safe and stress-free conditions for animals so that stress would not affect the results of experiments. During the induction of obesity, only two people responsible for the subsequent administration of the investigated compounds, were allowed to handle the animals. After experiment mice were killed by decapitation that is considered to be the most humane method of mouse euthanasia”.

3. We note that you have included the phrase “data not shown” in your manuscript. Unfortunately, this does not meet our data sharing requirements. PLOS does not permit references to inaccessible data. We require that authors provide all relevant data within the paper, Supporting Information files, or in an acceptable, public repository. Please add a citation to support this phrase or upload the data that corresponds with these findings to a stable repository (such as Figshare or Dryad) and provide and URLs, DOIs, or accession numbers that may be used to access these data. Or, if the data are not a core part of the research being presented in your study, we ask that you remove the phrase that refers to these data.

These data are not a core part of research being presented in our study. The phrase has been deleted. **LINE 179**

Reviewers' comments:

5. Review Comments to the Author

Please use the space provided to explain your answers to the questions above. You may also include additional comments for the author, including concerns about dual publication, research ethics, or publication ethics. (Please upload your review as an attachment if it exceeds 20,000 characters)

Reviewer #1: General Comments:

According to the World Health Organization (WHO), obesity has reached epidemic proportions globally. Obesity causes low-grade chronic inflammation which may lead to a number of chronic diseases. Additionally, A2 adenosine receptors play an important role in the regulation of the inflammatory process and glucose homeostasis.
Although caffeine, a non-selective A2A adenosine receptor antagonist, has been extensively studied by some research groups, the anti-inflammatory and anti-obesity mechanisms of some selective A2A adenosine receptor antagonists are few studied.
This paper reports the anti-inflammatory and anti-obesity activities of KD-64, a new selective A2A adenosine receptor antagonist, by using several current techniques and animal models.
The paper is original, well written and organized, and within the scope of Plos One.


Specific Comments

Title
The title should be concise. Authors should consider to reduce it.

We would like to leave it the way it is. In our opinion it contains the most interesting points of our investigation and in this way it might draw the attention of a potential reader.

Introduction
In page 5 – lines 106 and 107, it is referred that “…These compounds have similar structure to KD-64 – ligand we have used in the presented study…”.
KD-64 IUPAC chemical name must be included and the similarities between the chemical structures of KD-64 and the other referred compounds should be described.
Is KD-64 a caffeine analogue?

Introduction has been supplemented with the appropriate information. **LINES 102-103, 106-110, 111, 131-132, 161-162, Figure 1**

Experimental Design / Results and Discussion
Concerning the three animal models, more details must be included, namely:
• The age of the animals at the beginning of the experiences

Six-week old mice were used for both acute and obesity induction models. These information was added to manuscript in the Methods section. **LINES 136, 137**

• Considering that gender is a confounding variable, wouldn’t be better to use only one gender in all groups/models of the study? (Usually, male mice provide more pure and accurate responses…)

We agree that male mice provide more accurate responses therefore male mice were used in the acute models. However, in the obesity model, female mice were used for a specific reason.

Induction of obesity is difficult. It is desirable to get the largest differences between the weight of lean mice - standard control and the weight of obese mice - obese control. During induction of obesity the body weight of female mice stabilizes faster than that of males and in the following weeks females no longer gain weight. Males grow larger so their body weight changes longer. Since control female mice change little weight, while control obese mice quickly increase weight, in the model of obesity induced by the administration of high-fat feed a greater weight difference between these two groups can be obtained. When the differences in body weight are greater, the pharmacological effects of the tested compounds can be seen better.

• The concentration of caffeine used was much higher than the concentration of KD-64. Why? (This may explain, at least partially, the reported caffeine pro-inflammatory effect).

The caffeine dose of 50 mg/kg b.w./day, i.p. was selected based on the literature.

In studies on the effects of caffeine on body weight of obese mice, Wu et al., 2017 administered 60 mg/kg b.w./day, p.o. In studies on its analgesic effect in mice, Bach-Rojecky, 2003 used doses from 1.67 to 67 mg/kg b.w., i.p. Caffeine exerts a direct dose-dependent analgesic action. Pohanka, 2014, showed antioxidant activity of caffeine at the doses from 1 – 67 mg/kg b.w., i.m. Other study shows that, administration of a dose of 25 mg/kg b.w. to rats led to its anti-inflammatory effect [Li et al., 2011]. Caffeine at a dose of 100 mg/kg b.w. provided strong protection against acute liver damage, but at a dose of 25 mg/kg b.w. exacerbated liver damage. Ohta et al., 2007 showed that the effect of caffeine on inflammation is biphasically dependent on doses. Namely, lower doses of caffeine enhanced proinflammatory cytokine induction and tissue destruction, while a high dose of caffeine suppressed tissue damage by the inhibition of proinflammatory cytokine responses and the induction of an anti-inflammatory cytokine, IL-10.

As we wrote in the manuscript, caffeine administration has led to an increase in plasma IL-6 levels in some studies [Walker et al., 2007, Tauler et al., 2013]. In our study the same effect was observed. This does not mean, however, that caffeine has a pro-inflammatory effect.

Therefore it is difficult to answer the question whether the dose of 50 mg/kg b.w. could lead to the pro-inflammatory effects of caffeine.

Wu L, Meng J, Shen Q, Zhang Y, Pan S, Chen Z, Zhu LQ, Lu Y, Huang Y, Zhang G. Caffeine inhibits hypothalamic A1R to excite oxytocin neuron and ameliorate dietary obesity in mice. Nat Commun. 2017 Jun 27;8:15904. doi: 10.1038/ncomms15904.

Bach-Rojecky L. Analgesic effect of caffeine and clomipramine: a possible interaction between adenosine and serotonin systems. Acta Pharm. 2003 Mar;53(1):33-9.

Pohanka M. Caffeine alters oxidative homeostasis in the body of BALB/c mice. Bratisl Lek Listy. 2014;115(11):699-703.

Li XY, Xu L, Lin GS, Li XY, Jiang XJ, Wang T, Lü JJ, Zeng B. Protective effect of caffeine administration on myocardial ischemia/reperfusion injury in rats. Shock. 2011 Sep;36(3):289-94. doi: 10.1097/SHK.0b013e3182222915.

Ohta A, Lukashev D, Jackson EK, Fredholm BB, Sitkovsky M. 1,3,7-trimethylxanthine (caffeine) may exacerbate acute inflammatory liver injury by weakening the physiological immunosuppressive mechanism. J Immunol. 2007 Dec 1;179(11):7431-8.

Walker GJ, Finlay O, Griffiths H, Sylvester J, Williams M, Bishop NC. Immunoendocrine response to cycling following ingestion of caffeine and carbohydrate. Med Sci Sports Exerc. 2007;39:1554-1560.

Tauler P, Martinez S, Moreno S, Monjo M, Martínez P, Aguiló A. Effects of caffeine on the inflammatory response induced by a 15-km run competition. Med Sci Sports Exerc. 2013;45:1269–1276.

• Standard diet composition should be provided in a table (together with the high fat-diet composition)

Appropriate information has been added. **LINES: 241-249**

Reviewer #2: Dear Kotanska and colleagues,

I have some minor questions regarding your article:
- line 73-75 "the role of adenosine receptor signaling in the development and progressiona of numerous diseases has been emphasized for years" needs a reference.

The reference has been added and all subsequent literature renumbered. **LINE 75, 610-621**

Chen JF, Eltzschig HK, Fredholm BB. Adenosine receptors as drug targets--what are the challenges? Nat Rev Drug Discov. 2013 Apr;12(4):265-86. doi: 10.1038/nrd3955. Review.

- line 146: "in all experiments ketoprofenm caffeine or KD-64 were administered as suspensions in 1% Tween 80" in what solution? PBS? NaCL?

These compounds were administered in solution of 1% Tween 80 in sterile water for injection. Appropriate information has been added. **LINE 166 -167**

- line 258: how did you measure "spontanous activity"?

In this place of the manuscript I should have used wording „locomotor activity”. The sentence has been corrected. **LINE 297**

- figure 4 C in yy axis please add the wavelenght the absorbance was measured

The wavelength (620nm) has been added.
